# Parallel processing relies on a distributed, low-dimensional cortico-cerebellar architecture

**DOI:** 10.1162/netn_a_00308

**Published:** 2023-06-30

**Authors:** Eli J. Müller, Fulvia Palesi, Kevin Y. Hou, Joshua Tan, Thomas Close, Claudia A. M. Gandini Wheeler-Kingschott, Egidio D’Angelo, Fernando Calamante, James M. Shine

**Affiliations:** Complex Systems Research Group, The University of Sydney, Sydney, NSW, Australia; Brain and Mind Centre, The University of Sydney, Sydney, NSW, Australia; Brain Connectivity Research Center, IRCCS Mondino Foundation, Pavia, Italy; Department of Brain and Behavioral Sciences, University of Pavia, Pavia, Italy; National Imaging Facility, Sydney, NSW, Australia; School of Biomedical Engineering, The University of Sydney, Sydney, NSW, Australia; Sydney Imaging, The University of Sydney, Sydney, NSW, Australia; NMR Research Unit, Queen Square Multiple Sclerosis Centre, Faculty of Brain Sciences, UCL Queen Square Institute of Neurology, UCL, London, UK

**Keywords:** fMRI, Dual-task, Cerebellum, Cerebral cortex, Diffusion, Parallel

## Abstract

A characteristic feature of human cognition is our ability to ‘multi-task’—performing two or more tasks in parallel—particularly when one task is well learned. How the brain supports this capacity remains poorly understood. Most past studies have focussed on identifying the areas of the brain—typically the dorsolateral prefrontal cortex—that are required to navigate information-processing bottlenecks. In contrast, we take a systems neuroscience approach to test the hypothesis that the capacity to conduct effective parallel processing relies on a distributed architecture that interconnects the cerebral cortex with the cerebellum. The latter structure contains over half of the neurons in the adult human brain and is well suited to support the fast, effective, dynamic sequences required to perform tasks relatively automatically. By delegating stereotyped within-task computations to the cerebellum, the cerebral cortex can be freed up to focus on the more challenging aspects of performing the tasks in parallel. To test this hypothesis, we analysed task-based fMRI data from 50 participants who performed a task in which they either balanced an avatar on a screen (balance), performed serial-7 subtractions (calculation) or performed both in parallel (dual task). Using a set of approaches that include dimensionality reduction, structure-function coupling, and time-varying functional connectivity, we provide robust evidence in support of our hypothesis. We conclude that distributed interactions between the cerebral cortex and cerebellum are crucially involved in parallel processing in the human brain.

## Author Summary

How does the brain support the performance of multiple complex tasks, in parallel? The distributed architecture of the cerebellum is ideally placed to interact with the cerebral cortex, creating complex channels for segregated information processing that afford the execution of parallel tasks. Here, we apply time-resolved functional connectivity analyses to functional MRI data collected while individuals performed a dual task that required either balancing, calculating, or the two in tandem. We found robust evidence for distinct patterns of cortico-cerebellar connectivity as a function of task performance.

## INTRODUCTION

How do distributed whole-brain neural activity patterns give rise to human cognitive function? This question lies at the heart of modern psychology and neuroscience but, despite decades of neuroimaging experiments, we still do not have a clear answer. One reason is that conventional neuroimaging methods applied to data from cognitive tasks typically represent the brain as a static snapshot of independent parts or at best, ‘functionally connected’ pairs of brain regions (John et al., 2022). Another important issue is that neuroimaging experiments are usually designed to identify regions that are most selectively associated with a specific task, but are less well suited to distinguishing the presence of multiple concurrent cognitive constructs within the same task (Poldrack, 2012). For these reasons, many leading theories in cognitive neuroscience have relied on relatively static descriptions of the ‘key brain regions involved’ in a particular task.

In contrast to this view, there is evidence to suggest that the neural implementation of cognitive function in humans is far more dynamic and integrative (Eisenreich et al., 2017). In solving real world problems, we rarely isolate a specific cognitive capacity, such as focussed attention or resistance to distraction, but instead combine multiple cognitive constructs together in order to solve challenges in real time (Poldrack et al., 2011). Consider an experienced driver navigating heavy highway traffic in the pouring rain—the driver must remain focussed on the road, ensure the windshield wipers are on, regularly check their blind spots and also keep the pedals depressed at the appropriate level. This view of cognitive function in the real world is crucially dependent on the parallel processing of multiple distinct challenges; however, for the reasons outlined above, we still lack a satisfying description of how the human brain is capable of supporting parallel processing.

To facilitate complex coordinated behavioural responses underpinned by similarly complex spatiotemporal activity patterns, the brain may first learn to execute at least one of the computations automatically (i.e., without paying close, conscious attention to the completion of the task). To achieve this, the system must be capable of responding to specific contexts with a high degree of spatial and temporal precision (Schmitz & Duncan, 2018). Secondly, the responses must be relatively error free and reliable. Finally, the system must be able to be triggered in the presence of a specific stimulus or context without the need for deliberate attention. Without making the responses to different computational burdens relatively stereotyped in this fashion, performing two (or more) computations in parallel would require the prioritisation of one of the computations, likely to the detriment of the other task(s). In addition, any two tasks learned by the same network could potentially run into structural interference (Petri et al., 2021), particularly if the networks required to complete the overlapping tasks use similar cortical regions.

Crucially, the architecture of the cerebellum is ideally suited to fulfil each of the features required for automatic processing, both in the sensorimotor and cognitive domains (D’Angelo, 2019; D’Angelo & Casali, 2013; Ramnani, 2014; Shine et al., 2019). First, the cerebellum is organized in parallel modules with different cerebrocortical regions (D’Angelo & Casali, 2013). In direct contrast to the basal ganglia, the internal circuitry of the cerebellar cortex consists of sparse, distributed connectivity patterns that likely support dimensionality expansion (Cayco-Gajic & Silver, 2019), rather than reduction (as is the case for the basal ganglia; Bar-Gad et al., 2003; Wilson, 2013). In addition, the glutamatergic outputs of the cerebellum through the deep cerebellar nuclei innervate ‘core’ thalamic nuclei (Kuramoto et al., 2009), which project to the granular layers of the frontal cortex (Preuss & Wise, 2022) in a much more precise manner than the ‘matrix’ thalamic nuclei. There is also evidence that cerebellar circuits can condition on their own outputs, and hence learn to execute specific sequences of effects based on triggering context signals (Khilkevich et al., 2018). Anatomically, the cerebellum is bidirectionally interconnected with multiple cerebrocortical areas, with major tracts connecting the dentate nucleus to the frontal and prefrontal cerebral cortex, along with other associative areas (Palesi et al., 2015, 2017). Functionally, the cerebellum plays a critical role in shaping complex functional network dynamics (Palesi et al., 2020), as evidenced by its role in both resting-state (Castellazzi et al., 2014, 2018) and task-related neuroimaging studies (Alahmadi et al., 2016; Balsters & Ramnani, 2011; Casiraghi et al., 2019; Shine et al., 2019). Based on these architectural features and relationships with complex, dynamic neuroimaging patterns, we hypothesized that connections between the cerebellar cortex and cerebellum are crucial for the facilitation of parallel processing. Using a set of approaches that include dimensionality reduction, structure-function coupling, and time-varying functional connectivity, we provide robust evidence in support of our hypothesis.

## RESULTS

**Figure 1.**
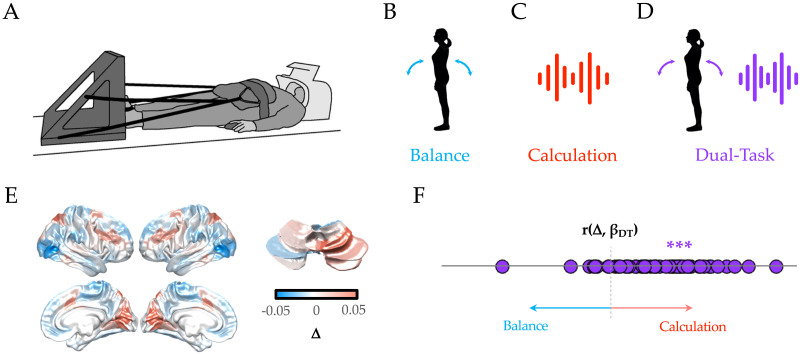
Low-dimensional balance between integration and segregation during dual-task performance. (A) participants lay supine in an MRI scanner, with their legs controlling a force plate. (B) Balance trials (blue) involved a dynamically moving avatar that the participant had to match. (C) calculation trials involved listening to a series of beeps, and then subtracting the multiple of 7 times the number of beeps from a cue number (red). (D) dual-task trials required performing both tasks, simultaneously (purple). (E) The calculation trials recruited increased BOLD in fronto-parietal and visual cortices, along with right superior cerebellum, whereas balance trials were associated with increased BOLD in lateral visual cortex, medial motor cortex, and parietal operculum. (F) the dual-task β map across all 50 subjects was more similar to the calculation β map (i.e., positive correlation with λ1) than the balance β map (i.e., inverse correlation with λ1); *** *p* < 0.001.

To test this hypothesis, we reanalysed an existing fMRI dataset (Papegaaij et al., 2017) consisting of 50 healthy individuals dual task in a 3T MRI scanner with their feet resting on a force plate (Figure 1A), and their vision oriented towards a two-dimensional avatar that tilted forward and backward. There were three distinct trial types: during balance blocks (Figure 1B, blue), participants had to stabilize the slow fluctuations of the avatar using forward and backward movements on the force plate; during calculation blocks (Figure 1C, red), subjects had to track between three and four audible beeps, and then subtract that number, multiplied by 7, from a cue number presented prior to the trial; and during dual-task blocks (Figure 1D, purple), subjects performed both trials simultaneously.

### Brain State Signatures During Dual-Task Performance

First, we compared the BOLD patterns associated with the performance of the three different tasks blocks. Specifically, we created a difference map between the average group-level β parameters estimated from 400 cortical and 28 cerebellar regions of interest for the balance and calculation trials (Δ) (Figure 1E). By comparing this difference map to the β map from the dual-task trials—r(Δ, βDT)—we could determine whether performing the two tasks in tandem led to a brain map that was more or less like one or the other single tasks—a positive correlation with this map (λ1) was suggestive of the dual task reflecting the more challenging calculation task, a negative correlation with the less challenging balance task, and a null correlation with the notion of optimally splitting activity between the two (or a pattern orthogonal to the two single tasks). Consistent with the second option, we found that the low-dimensional signature of dual-task performance was more similar to the calculation β map than the balance β map (*r* = 0.192 ± 0.05, *p* = 6.5 × 10^−5^; Figure 1F), suggesting that during the dual-task trials, the cerebral cortex and cerebellum configured their activity to ensure the effective completion of the calculation trials.

Despite the brain states during dual-task trials having more in common with the calculation than the balance trials, close examination of the RMS error of the balance portion of the dual-task trials suggests that subjects were performing the task as well as than when they performed the balance trial on its own (Kolmogorov–Smirnov test: *p* = 0.358). So how was the brain configured on these dual-task trials in order to mediate this stability? Based on previous empirical (Balsters & Ramnani, 2011) and theoretical (D’Angelo & Casali, 2013; Shine, 2020; Shine & Shine, 2014) work, we hypothesized that the distributed architecture integrating the cerebral cortex and cerebellum should be important for mediating this putative parallel processing performance. One straightforward prediction is that balancing multiple tasks at the same time should recruit more regions of the cerebellum, and hence that cerebellar blood flow should be more strongly associated with dual-task performance than either the balance or calculation task alone. We found evidence to confirm this hypothesis—namely, greater proportion of cerebellar regions were associated with a positive mean β value in dual task as compared to balance and/or calculation trials (67.3% vs. 35.7% and 39.3%, respectively; χ2 (2, *N* = 50) = 249.6, *p* < 1.0 × 10^−4^).

### Unique Patterns of Cortico-Cerebellar Functional Connectivity During Dual-Task Performance

**Figure 2.**
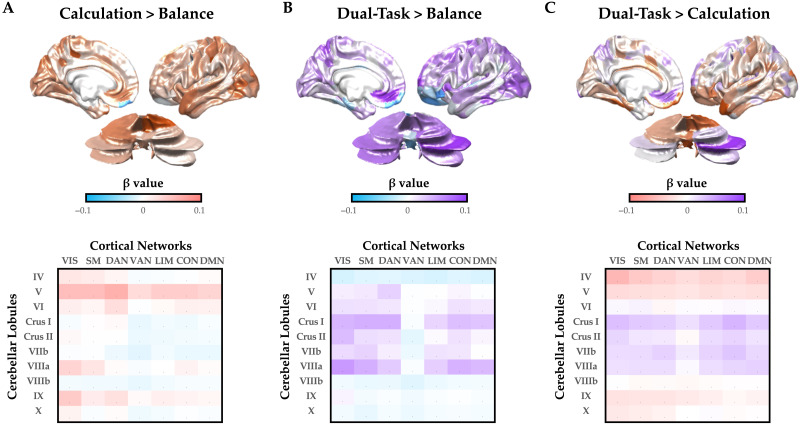
Cortico-cerebellar task-based functional connectivity. (A) patterns of task-based cortico-cerebellar functional connectivity during calculation (red) when compared to balance (blue) trials—upper: mean task-based connectivity strength for cerebral cortex and cerebellum; lower: mean task-based connectivity strength collapsed into 7 Yeo networks (columns) and 10 average lobules (rows). (B) similar maps for dual task (purple) versus balance. (C) similar maps for dual task versus calculation. Note: results were consistent for left and right hemispheres. VIS = visual; SM = somatomotor; DAN = dorsal attention network; VAN = ventral attention network; LIM = limbic network; CON = control network; DMN = default mode network.

Given that the dual-task trials were more similar to calculation trials than balance trials (Figure 1F), how was the brain capable of supporting multiple tasks at the same time? We hypothesized that balance, calculation, and dual-task trials should have unique patterns of cortico-cerebellar functional connectivity that could allow the brain to support multiple channels of communication within the same system. To test this hypothesis, we calculated the time-varying functional connectivity between all cortical and cerebellar parcels using the Multiplication of Temporal Derivatives approach (window = 20 TRs; Shine et al., 2015) and then contrasted the three trial types with one another. We observed robust differences between the three trial types (Figure 2). For instance, calculation trials (when compared to balance trials) were associated with widespread cortico-cerebellar connectivity between lobule V and the majority of cortical networks, as well as more targeted connections between VIIIa/IX and primary sensorimotor networks (Figure 2A). In contrast, balance trials (when compared to calculation trials) showed predominant increases in intermediate cerebellar lobules (e.g., Crus I and II) with higher order cortical networks. In contrast, dual-task trials were associated with heightened fronto-parietal connections with intermediate cerebellar lobules, particularly Crus I and VIIIa, when compared to both balance (Figure 2B) and calculation trials (Figure 2C).

**Figure 3.**
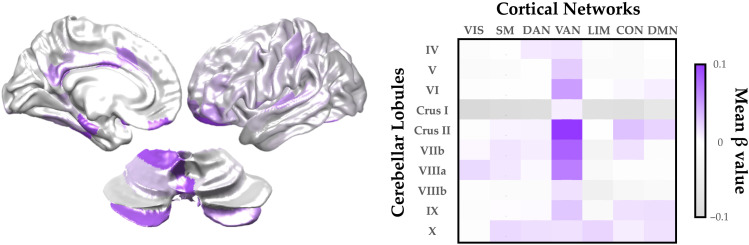
Increased cortico-cerebellar task-based functional connectivity associated with successful dual-task performance. Left: Key cortical and cerebellar regions with heightened task-based functional connectivity during dual-task trials with correct versus incorrect answers. Right: Mean significant β-value (cortical sub-network [Yeo 7 atlas] vs. cerebellar lobule [SUIT atlas]) associated with task-based functional connectivity values for correct versus incorrect dual-task performance (*p* < 0.001; random permutation test). VIS = visual; SM = somatomotor; DAN = dorsal attention network; VAN = ventral attention network; LIM = limbic network; CON = control network; DMN = default mode network.

Having confirmed a robust relationship between the cerebral cortex and cerebellum during dual-task performance, we next asked whether cortico-cerebellar functional connectivity patterns differentiated between correct and error dual-task trials. To test this hypothesis, we fit a General Linear Model to each dual-task trial, independently, for each cortico-cerebellar time-varying connectivity score. We then split dual-task trials into correct (accurate calculation and small RMS error [<50% of population distribution]) and incorrect (inaccurate calculation, large RMS error [>50%] or both) trials and compared (using a set of independent-samples *t* tests) the task-based functional connectivity between cortical and cerebellar parcels as a function of effective dual-task performance. We conducted a permutation test (5,000 iterations) to determine the likelihood of each edge being distinct between the two groups by chance. To summarize these results, we computed the mean significant β-value for the functional connectivity between each cerebellar lobule (averaged across hemispheres, and ignoring the connections of the vermis; from the cerebellar SUIT atlas (Diedrichsen, 2006)) and each of 7 pre-identified cortical networks (the Yeo 7 parcellation from the 400-region Schaefer atlas (Schaefer et al., 2018; Figure 3). We found a robust increase in task-based functional connectivity between the ventral attention network (VAN) and lobules Crus II, VIIb, VIIIa and VI (Figure 3), as well as more distributed connections between lobule X and multiple cortical subnetworks. In contrast, Crus I was relatively functionally disconnected from all cortical networks (except VAN) during effective dual-task performance, which is consistent with known patterns of cerebellar lesion-related cognitive impairments (Ilg et al., 2013).

### Dual-Task Performance Balances Network Integration and Segregation

**Figure 4.**
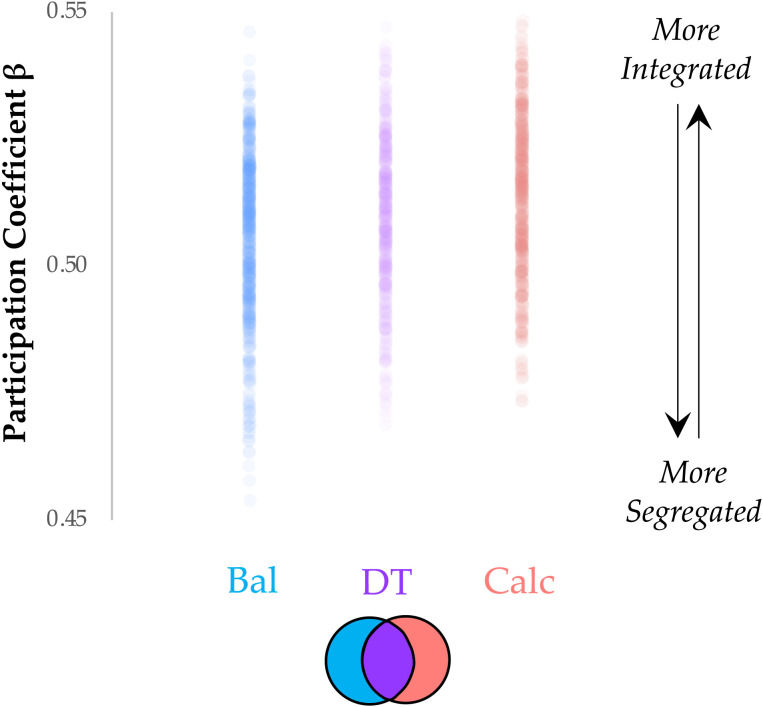
Parallel processing balances integration and segregation. Balance trials were associated with relative segregation (low PC; blue), calculation trials with relative integration (high PC; red), and dual-task trials with a balance between integration and segregation (intermediate PC; purple); *F*2,147 = 3.41; *p* = 0.036. Thick lines represent the median value for each group.

One way in which the distributed cortico-cerebellar architecture could facilitate effective parallel processing is by striking an effective balance between integration and segregation (Bassett et al., 2015, p. 201; Mohr et al., 2016; Shine & Poldrack, 2017). In previous work, we have used a combination of time-varying functional connectivity and a topological measure that quantifies network-level integration—the participation coefficient (PC; Shine et al., 2016)—to demonstrate that the systems-level network structure of functional connectivity changes during task performance, with cognitively challenging tasks requiring higher integration than relatively simple tasks (Shine et al., 2016). From this, we predicted that the balance task should be relatively segregated (i.e., low PC), the calculation task should be relatively integrated (i.e., high PC), and the dual-task trials should strike a balance between the two extremes (i.e., intermediate PC). Using our standard time-varying analysis (see Methods), we observed robust evidence for our predictions (Figure 4; *F*2,147 = 3.41; *p* = 0.036). In addition, although the dual-task topological pattern was positively correlated with the average of balance and calculation (*r* = 0.464; *p* < 0.001), it was not a direct superposition of the two maps, suggesting topological reconfiguration during the different task states. Together, these results confirm that parallel processing in the brain is supported by a topological balance between integration and segregation.

### Cortico-Cerebellar Activity Flow Mapping

**Figure 5.**
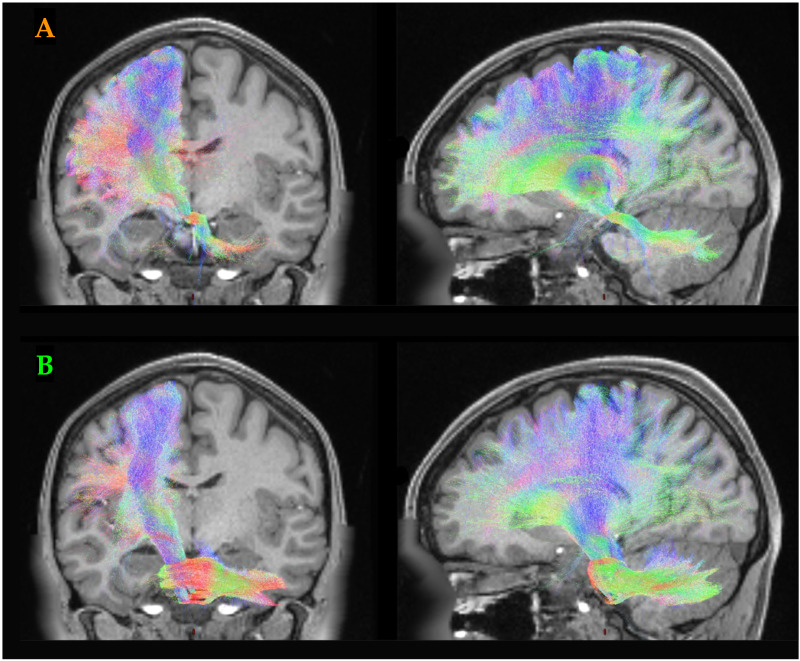
White matter streamlines interconnecting the cerebellum and the cerebral cortex. (A) the cortico-ponto-cerebellar (CPC) tract sends projections from the cortex via the pontine nuclei into the mossy fibres of the cerebellum; (B) the cerebello-thalamo-cortical (CTC) tract derives from the deep cerebellar nuclei, which project back via the core thalamic nuclei to the cerebral cortex. Tracts created using mrtrix were projected onto a T1-weighted structural image from individual 100307 from the Human Connectome Project (de-faced to preserve autonomy). The colours of each tract represent the primary direction of each tract: blue = inferior-superior; red = left-right; green = anterior-posterior.

The input and output streams of cerebral cortex and cerebellum interact via distinct white matter pathways. Importantly, while the structural connections between these two structures are reciprocal, they are imbalanced (Palesi et al., 2015, 2017)—different pathways exist from the cerebral cortex to the cerebellum than from the cerebellum to the cerebral cortex. Specifically, thick-tufted layer V pyramidal neurons in the deep layers of the cerebral cortex send projections to the mossy fibre pathway of the cerebellum (via the pontine nuclei), thus forming the cortico-ponto-cerebellar (CPC) tract (Figure 5A). In contrast, the cerebral cortex receives feedback from the cerebellum via the deep cerebellar nuclei, which project via the ‘Core’ nuclei of the thalamus—that is, the cerebello-thalamo-cortical (CTC) tract (Figure 5B). Plastic changes between the mossy fibre pathway and the Purkinje cells of the cerebellar cortex are proposed to act as a major site for the refinement of automatized behaviour (D’Angelo et al., 2016; Ramnani, 2014; Shine, 2020; Shine & Shine, 2014) and hence, the capacity to perform multiple tasks simultaneously. From our observations that the time series of the cerebral cortex and cerebellum were highly coordinated during dual-task behaviour, we hypothesized that the specific patterns of BOLD activity in both the cortex and cerebellum should be related to the intersection between prior BOLD activity in the cerebellum (via the CTC) and cerebral cortex (via the CPC).

**Figure 6.**
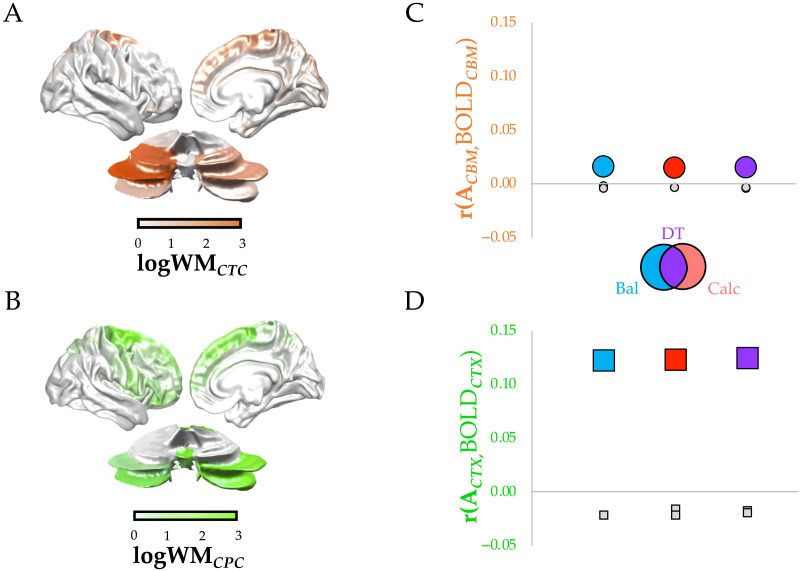
Cortico-cerebellar structure-function mapping across trial types. (A) normalized (in log10 of white matter connectivity) map of projections from the cerebral cortex to cerebellum via CPC (orange). (B) normalized (in log10 of white matter connectivity) map of projections from cerebellum to the cerebellar cortex via CTC (green). (C) activity flow mapping (Cole et al., 2016) between cerebellar BOLD patterns predicted from CPC tract in balance (Bal, blue), calculation (Calc, orange), and dual-task (DT, purple) trials (circles); see Methods for details. (D) the same for cortical BOLD patterns predicted from the CTC tract (squares). All activity flow map correlations were greater than permuted null levels.

To test this hypothesis, we adapted the activity flow mapping approach (Cole et al., 2016) to incorporate the structural connectivity between the cortex and cerebellum. Specifically, we extracted 9 × 107 structural connectivity weights for both the contralateral CPC (Figure 6A, orange) and CTC (Figure 6B, green) tracts (Palesi et al., 2017) from a single healthy 26–30-year-old female (ID no. 100307) from the Human Connectome Project (a single subject connectome was chosen so as to retain precision in the parcel-to-parcel connectivity estimates for both CPC and CTC—note, however, that maps were highly similar to those previously extracted from 28 healthy participants from the HCP (Palesi et al., 2017)). While both tracts are overexpressed in the frontal cortices, there were relatively more CPC projections from the parietal lobes and more CTC projections that innervate the frontal cortex, which is consistent with known anatomical projection patterns (D’Angelo & Casali, 2013; Prevosto & Sommer, 2013; Ramnani, 2006; Shine, 2020). A parsimonious interpretation of these data is that the frontal cortex benefits from the information provided to the cerebellum by posterior cortices that process potential opportunities for action (also known as affordances; Pezzulo & Cisek, 2016).

If cortico-cerebellar communication is required for effective dual-task performance, then blood flow within either the cerebral cortex or cerebellum during dual-task trials should be predictive of subsequent blood flow (assuming sufficient delay) within the cortical (or cerebellar) regions to which they are connected by white matter projections. To create an estimate of what these predicted BOLD responses should be, we created two template maps—one for predicted cerebellar activity (estimated cerebellar activity: ACTX = WCBM . CPC) and one for predicted cortical activity (estimated cortical activity: ACBM = WCTX . CTC)—by multiplying the cortico-cerebellar structural connectivity matrices with the preprocessed BOLD pattern observed during the three different trial types. We then correlated these prediction vectors with the actual BOLD patterns in the respective regions. If the observed patterns of activity were similar, we can conclude that BOLD activity patterns were intimately related to the reciprocal structural connections between the cerebral cortex and cerebellum.

Across all three trial types, both cortico-cerebellar (via CPC; Figure 6C, circles) and cerebello-cortical (via CTC; Figure 6D, squares) activity flow patterns were significantly greater for actual versus randomly shuffled data (all *p* < 0.05), suggesting that functional activity was coordinated by connections both from the cerebral cortex to the cerebellum (i.e., CPC) and vice versa (i.e., CTC) across all tasks. Interestingly, despite the consistent positive relationships, cerebello-cortical connections (i.e., CTC) were more robustly able to predict subsequent cortical patterns than cortico-cerebellar connections (i.e., CPC), suggesting that the feedback from the cerebellum to the cerebral cortex was more crucial for task performance. Finally, we found that the match between ACTX/ACBM and the raw data was greater in correct versus incorrect dual-task trials for both cerebral cortex (T = 2.397, *p* = 0.017) and cerebellum (T = 2.049, *p* = 0.041), further confirming the importance of cortico-cerebellar interaction for parallel processing.

## DISCUSSION

In this study, we used systems-level neuroimaging analysis to demonstrate that robust interactions between the cerebral cortex and cerebellum are associated with effective dual-task performance. We hypothesized that, through distributed white matter pathways that interconnect these major cortical systems, the brain can differentiate different task contexts so as to effectively maintain the performance of two computational tasks in parallel. To test this hypothesis, we analysed BOLD data from the cerebral cortex and cerebellum, and in doing so demonstrated that dual-Task performance recruited heightened cerebellar activity (Figure 1) and functional connectivity between the cerebral cortex and cerebellum (Figures 2 and 3) that was linked to the balance between integration and segregation (Figure 4) and related to the structural connections between the cerebellum and cerebral cortex (Figures 5 and 6). Together, these results highlight the importance of systems-level interactions in the manifestation of complex cognitive capacities.

Our results clearly demonstrate that models that incorporate the cerebellum and its massive, high-dimensional architecture provide a more parsimonious account for how the brain can balance the challenges inherent with parallel processing (Balsters & Ramnani, 2011; D’Angelo & Casali, 2013; Shine, 2020; Wu et al., 2013). The distributed circuits that interconnect the cerebral cortex and cerebellum are optimally set up to fulfil this capacity. Specifically, the major output of the cerebral cortex—layer V PT-type pyramidal neurons—provides the primary afferent input to the cerebellar cortex (i.e., granule cells), by way of the pontine nuclei (D’Angelo & Casali, 2013; Kratochwil et al., 2017; Shine, 2020). Following a massive dimensionality expansion that has been argued to facilitate pattern separation (Cayco-Gajic & Silver, 2019), the outputs of the cerebellum (the deep cerebellar nuclei) send large glutamatergic projections to the ventral tier of the thalamus (Prevosto & Sommer, 2013), wherein they innervate the cerebral cortex. The thalamic targets of the cerebellum then go on to drive activity in the cerebral cortex, typically in a high-frequency, precise fashion (Nashef et al., 2022) that we have argued form the basis of relatively automatic modes of behaviour (Shine, 2020; Shine & Shine, 2014). Here, we extend these functional neuroanatomical principles to incorporate the completion of challenging dual tasks, thus augmenting and reinforcing conclusions from previous functional neuroimaging work on dual-task performance (Balsters & Ramnani, 2011; Shine & Poldrack, 2017; Wu et al., 2013). We anticipate that similar patterns will be observed in future experiments that interrogate different types of dual tasks, particularly those in which one (or both) of the tasks is capable of relative automaticity. Whether such automaticity benefits extend to purely perceptual tasks, such as the attentional blink (Sergent & Dehaene, 2004), is an interesting open question for future work.

The topological signature of functional networks estimated from BOLD data have previously been linked to effective performance on cognitive tasks. For instance, an integrated brain has been linked to the completion of a range of complex tasks, such as those that probe working memory (Cruzat et al., 2018; Fransson et al., 2018; Shine et al., 2016), logical reasoning (Hearne et al., 2017), and attentional tracking (Mäki-Marttunen, 2021; Wainstein et al., 2021). In contrast, a relatively segregated functional network has been linked to relative sensorimotor automaticity (Bassett et al., 2015; Mohr et al., 2016), as well as to attentional vigilance (Sadaghiani et al., 2015). Our results are consistent with the spectrum implied by these previous results—the balance task, which presumably tapped into relatively well-learned behaviours, was associated with a segregated functional network; and the calculation task, which likely required more focussed, flexible attention, was associated with a relatively integrated network. Interestingly, although the dual-task trials were arguably more challenging than the calculation trials on their own, the topology of the network actually demonstrated a balance between integration and segregation, suggesting that performing tasks in parallel requires an ability to avoid topological extremes, perhaps so as to maximise information-processing capabilities (Sporns, 2013). In addition, there are theoretical reasons to believe that the finite nature of biological networks may imbue specific limits on the number of possible tasks that can be run in parallel, although we expect that the high-dimensional architecture of the cerebellum (Cayco-Gajic & Silver, 2019) will likely boost this capacity, particularly as a function of experience (Shine, 2020; Shine & Shine, 2014). Precisely which systems in the brain help to control this balance remains an open question; however, there are intriguing results that suggest that the neuromodulatory system may play a crucial role in this process (Breton-Provencher et al., 2022; Shine, 2020; Shine et al., 2021).

Systems-level neuroimaging analysis provides an integrated perspective of cognitive capacities; however, BOLD dynamics are necessarily indirect, that is, they don’t measure neural activity directly, but rather filtered through the low-dimensional lens of perfusion (Aquino et al., 2014; Pang et al., 2016). While the BOLD signal remains a robust measurement for neural signalling (Attwell & Iadecola, 2002; Moore & Cao, 2008), it only reveals a part of how the brain functions. This is particularly true for the cerebellar cortex, whose complex, convoluted anatomy (Caligiore et al., 2016; D’Angelo & Casali, 2013) and idiosyncratic firing properties (Khilkevich et al., 2018; Kostadinov et al., 2019; Person & Raman, 2011) render simple, linear readouts of neural activity from BOLD problematic. Specifically, there is evidence to suggest that BOLD measurements in the cerebellar cortex predominantly track activity in the mossy fibre pathway (via the CPC; Caesar et al., 2003; Mathiesen et al., 2000), whereas outputs from the Purkinje cells (via the CTC) are more difficult to characterize with BOLD signalling (Diedrichsen et al., 2019; Thomsen et al., 2009). While this does suggest caution with respect to the interpretation of our results, it makes the presence of robust cerebello-cortical activity flow mapping via the CTC (Figure 6D) all the more fascinating of a result, as it suggests that the fate of the Purkinje cells is relatively sealed by the specific pattern of mossy fibre inputs that they received, although we anticipate that this mapping is likely augmented by the process of learning, that is, it should be less profound when facing highly novel task contexts. Irrespectively, we hope that by consolidating analysis from multiple neuroimaging techniques, we have provided a robust illustration of changes to cortico-cerebellar circuits during a parallel processing task.

The capacity to perform tasks in parallel clearly scales positively with experience. In the future, it will be fascinating to examine the interactions between the cerebral cortex and cerebellum as individuals learn to perform individual tasks to relative automaticity. There is robust empirical previous work linking cerebellar output with highly overtrained behaviours in rodents (Callu et al., 2013). Similar arguments have been made when analysing automaticity in the performance of challenging cognitive tasks (Balsters & Ramnani, 2011). Interestingly, there is also evidence suggesting that, over the course of learning a simple sensorimotor task, the brain shifts from a relatively integrated to a segregated architecture (Bassett et al., 2015; Mohr et al., 2016). This suggests a novel prediction: the extent to which a particular task has been well learned will lead to relative segregation of the topological network signature of the brain, which in turn will make the task easier to automatise, and hence to combine successfully with other, more challenging dual tasks.

One factor that was not well controlled in this study was cognitive load, which is known to play an important role in our capacity to perform multiple tasks in parallel (Just et al., 2001; Michael et al., 2001; Whelan, 2007). Simply put, it is much easier to perform two tasks simultaneously if (at least) one of the tasks is either highly automatic or is sufficiently easy that its performance requires little to no focussed attention (Fischer & Plessow, 2015). In these cases, the simpler or more automatic task can be performed with minimal awareness, freeing up higher cognitive systems to aid in the completion of the second, harder/more challenging task. In our study, the balance task was presumed to be easier than the calculation task, as participants were expected to have been unlikely to have practiced the subtraction of the digit “7” from random large numbers, whereas balance is something many of us perform so much as to take it for granted. In future studies, it will be important to attempt to stack together multiple tasks that are difficult to perform in the same manner, such as comprehending an auditory stream while performing a calculation on concurrent visual input. Although we anticipate that both the cerebellum and cerebral cortex would be engaged in such a task, it is less likely that effective performance would be as crucially dependent on their interaction, as the mechanism we propose invokes the cerebellar-mediated anticipation of expected consequences as a means for freeing up higher cognitive resources (Ramnani, 2014; Shine, 2020; Shine & Shine, 2014). It is not currently clear whether these anticipatory processes are as important in the more deliberate, flexible stages of cognitive processing that would be required to complete two more deliberate cognitive tasks simultaneously.

Here, we have demonstrated that dynamic interactions between the cerebral cortex and cerebellum are critically related to the performance of a challenging dual task. Future research is required to determine whether similar principals are related to parallel processing of other simultaneous cognitive and perceptual challenges, as well as across distinct spatiotemporal scales.

## METHODS

### Experimental Setup

The functional data from this study arose from a re-analysis of a previously published dataset (Papegaaij et al., 2017); here, we will include the minimal information required to interpret the results, and point the interested reader to the original study for full details. 50 healthy female participants (mean age = 49 ± 20 years; Papegaaij et al., 2017) lay supine in the MRI scanner with their feet against a custom-made force platform attached to the MRI bed (Figure 1A; sample frequency of 100 Hz), with the position of the force platform was adjusted to subject height. To minimize excessive head movement, participants were pulled towards the force platform using thick elastic ropes attached to a hip belt (Papegaaij et al., 2017). A four-button device was placed underneath the right hand for the calculation task. The tasks were projected onto a white screen placed at the head of the scanner. Participants could see the screen via a mirror attached to the head coil.

During the balance task, an avatar in the shape of a woman was displayed on the screen. The avatar swayed forward and backward. Participants were instructed to try to keep the avatar in the upright position by increasing or decreasing the level of plantar flexion force measured by the load cell. As in normal standing, increasing the plantar flexion force led to a backward sway (and v.v.). At the start of every balance condition, participants were given 2 seconds to bring the avatar in the upright position. After these 2 seconds, a disturbance signal was added, causing the avatar to sway forward and backward. To keep the avatar upright, participants had to counteract these disturbances. The disturbance signal was made by combining 15 sinusoidal signals with random phases and with frequency characteristics based on an average frequency spectrum of centre of pressure movement during upright standing (0.025–1 Hz), measured in 10 young and 10 old adults. The maximum amplitude of the disturbance was ±30°. The error for each balance trial was created by calculating the sum of the root-mean-squared error between the optimal balanced avatar (i.e., 900) and the position of the actual avatar. Trials were subsequently median split to identify ‘good’ and ‘bad’ balance trials.

The calculation task consisted of serial subtractions with increments of seven—at the beginning of each trial, a number between 50 and 100 was projected on the screen for 2 seconds, after which a plus sign was displayed on the screen and a beep was generated every 3 to 4 seconds through an MRI compatible headphone (MR confon Optime 1, Magdeburg, Germany), with a total of four beeps per trial. Participants were instructed to subtract the number 7 with every beep. At the end of each trial, four answer possibilities were displayed on the screen: one indicating the correct answer, two erroneous answers, and the option that none of the other answers is correct. Participants indicated which answer they thought was correct by pressing the corresponding button of the four-button device.

During the dual-task condition, subjects performed the balance and calculation tasks simultaneously. The distribution of RMS errors in the balance trials and dual-task trials were compared using a Kolmogorov–Smirnov test.

An fMRI block design was used to alternate between the three conditions: balance, calculation, and dual task. Every participant performed 12 blocks, each block including one trial of each condition (thus three trials), with the order of the conditions randomized, both across blocks and between subjects. At the end of every block a 15-second rest period was given in which the participants fixated their gaze on a plus sign.

### MRI Acquisition and Preprocessing

Brain imaging was performed on a 3-T SIEMENS Magnetom Skyra System (Siemens, Erlangen, Germany) with a 20-channel head/neck coil. For functional scans, a T2*-weighted multiband gradient echo-planar imaging sequence was used (TR = 700 msec, TE = 30 msec, flip angle = 55°, 48 axial slices, slice thickness = 3 mm, no gap, in-plane resolution 3 × 3 mm) (Feinberg et al., 2010). After the functional scanning session, a high-resolution magnetization-prepared rapid acquisition gradient echo (MPRAGE) T1-weighted sequence (TR = 2,100 msec, TE = 4.6 msec, TI = 900 msec, flip angle = 8°, 192 contiguous slices, voxel resolution 1 mm^3^, FOV = 256 × 256 × 192 mm, iPAT factor of 2) was obtained in sagittal orientation. These anatomical scans were used to coregister the functional runs using SPM 12. The anatomical scan was segmented using the SPM tissue probability maps. The functional data were preprocessed as part of a different study (Papegaaij et al., 2017). For each subject, interscan movement was corrected by realigning and unwarping the data, with the first scan as a reference. All functional scans were then coregistered to the anatomical scan and normalized to the Montreal Neurological Institute (MNI) template brain via the forward deformations revealed by the structural segmentation. Movement in the scanner was assessed by calculating framewise displacement (FD) from the derivatives of the six rigid body realignment parameters estimated during standard volume realignment, as well as the root-mean-square change in BOLD signal from volume to volume (aka DVARS). Across the cohort, head motion was found to be minimal (group mean FD = 0.183 ± 0.08 mm; group mean DVARS = 0.811 ± 0.13).

Temporal artifacts were identified in each dataset by calculating FD from the derivatives of the six rigid body realignment parameters estimated during standard volume realignment (Power et al., 2014), as well as the root-mean-square change in BOLD signal from volume to volume (DVARS). Frames associated with FD > 0.25 mm or DVARS > 2.5% were identified; however, as no participants were identified with greater than 10% of the resting time points exceeding these values, no trials were excluded from further analysis.

### Brain Parcellation

Following preprocessing, the mean time series was extracted from 400 predefined cortical parcels using the Schaefer atlas (Schaefer et al., 2018) and 28 predefined cerebellar parcels from the SUIT atlas (Diedrichsen, 2006) (cerebellar nuclei were not included). The mean BOLD signal intensity from each region was extracted and then used for subsequent analyses.

### General Linear Model and Principal Component Analysis

A general linear model was fit to preprocessed, parcellated BOLD data with separate terms modelling each trial type (i.e., balance, calculation, and dual task). The event time series used to analyse the task included a convolution with a canonical haemodynamic response function. The proportion of cerebellar regions associated with positive cerebellar β-values was compared across balance, calculation and dual-task trials using a χ2 test with degrees of freedom = (rows − 1) × (columns − 1) = (3 − 1) × (2 − 1) = 2.

The average β-value for the balance and calculation trials were demeaned and analysed with a principal component analysis. The coefficient of the leading principal component was correlated with the mean β map from the balance and calculation trials to demonstrate its utility as a linear decoder between balance and calculation. The dot product between the dual-task β map for each subject and the leading principal component was calculated, and then subjected to a one-sample *t* test to determine whether the loading was more similar to calculation (positive loadings) or balance (negative loadings).

### Time-Varying Functional Connectivity

We used the multiplication of temporal derivatives (MTD) approach (Shine et al., 2015) to calculate time-resolved dynamic functional connectivity between the selected ROIs; code is freely available at https://github.com/macshine/coupling/ with a window size of 20 TRs (results were stable for window sizes of 10–50 TR). For each node, *n*, with time points, *t*, a vector of *t* − 1 temporal derivatives was calculated and normalized (temporal derivatives divided by the standard deviation of temporal derivatives, σ). Then, we created a matrix of functional coupling between the *i*th and *j*th nodes for each time point, by multiplying the temporal derivatives of each pair of nodes across each time point.

$${MTD}_{ijt}=\frac{1}{w}\sum_{t}^{t+w}\frac{\left({dt}_{it}\times {dt}_{jt}\right)}{\left(\sigma_{{dt}_{i}}\times \sigma_{{dt}_{j}}\right)}$$ (1)

where *dt* is the first temporal derivative of the *i*th and *j*th time series, and σ standard deviation of the temporal derivative, *w* is the window length of the simple moving average (Shine et al., 2015). The MTD values for the cortico-cerebellar system (i.e., 400 × 28 = 11,200 edges) were entered into a similar general linear model to the cortico-cerebellar BOLD values, with a permutation test (5,000 iterations) used to test for statistical significance.

### Modularity Maximization

The Louvain modularity algorithm from the Brain Connectivity Toolbox (BCT; Rubinov & Sporns, 2010; https://www.brain-connectivity-toolbox.net) was used on the neural network edge weights to estimate community structure. The Louvain algorithm iteratively maximizes the modularity statistic, Q, for different community assignments until the maximum possible score of Q has been obtained (see Equation 2). The modularity of a given network is therefore a quantification of the extent to which the network may be subdivided into communities with stronger within-module than between-module connections.

$$Q_{T}=\frac{1}{\upsilon^{+}}\sum_{ij}\left(w_{ij}^{+}-e_{ij}^{+}\right)\delta_{M_{i}M_{j}}-\frac{1}{\upsilon^{+}+\upsilon^{-}}\sum_{ij}\left(w_{ij}^{-}-e_{ij}^{-}\right)\delta_{M_{i}M_{j}}$$ (2)

where *v* is the total weight of the network (sum of all negative and positive connections), *w*_*ij*_ is the weighted and signed connection between regions *i* and *j*, *e*_*ij*_ is the strength of a connection divided by the total weight of the network, and δ_*M*_*i*_*M*_*j*__ is set to 1 when regions are in the same community and 0 otherwise; + and − superscripts denote all positive and negative connections, respectively.

For each epoch, we assessed the community assignment for each region 500 times and a consensus partition was identified using a fine-tuning algorithm (BCT). We calculated all graph theoretical measures on unthresholded, weighted, and signed connectivity matrices (Rubinov & Sporns, 2010). The stability of the γ parameter was estimated by iteratively calculating the modularity across a range of γ values (0.5–2.5; mean Pearson’s *r* = 0.859 ± 0.01) on the time-averaged connectivity matrix for each subject—across iterations and subjects, a γ value of 1.0 was found to be the least variable, and hence was used for the resultant topological analyses.

### Participation Coefficient

The participation coefficient, PC, quantifies the extent to which a region connects across all modules (i.e., between-module strength) and has previously been used to successfully characterize hubs within brain networks (Shine et al., 2016, 2019). The PC for each region was calculated within each temporal window using Equation 3, where *k*_*isT*_ is the strength of the positive connections of region *i* to regions in module *s* at time *T*, and *k*_*iT*_ is the sum of strengths of all positive connections of region *i* at time *T*. Negative connections were discarded prior to calculation. The PC of a region is therefore close to 1 if its connections are uniformly distributed among all the modules and 0 if all of its links are within its own module.

$$\text{PC}=1-\sum_{s=1}^{n_{M}}{\left(\frac{\kappa_{isT}}{\kappa_{iT}}\right)}^{2}$$ (3)

The PC for each parcel was compared across balance, calculation and dual-task trials using paired *t* tests (FDR *q* = 0.05).

### Diffusion MRI Analysis

Data were selected from a single 26–30-year-old female subject from the HCP (code: 100307). The minimally processed HCP diffusion dataset (which included correction for motion, susceptibility distortions, gradient nonlinearity and eddy currents) were subject to additional image processing, which multishell multitissue constrained spherical deconvolution to generate the fibre orientation distribution (FOD) in each voxel (Jeurissen et al., 2014; Tournier et al., 2004, 2007). These steps were implemented in accordance with previous work (Civier et al., 2019) and were performed using the MRtrix software package (https://www.mrtrix.org; Tournier et al., 2012, 2019).

The T1-weighted images were used to generate a so-called ‘five-tissue-type’ (5TT) image (R. E. Smith et al., 2012) using FSL (S. M. Smith et al., 2004); the 5TT image classifies the voxel into one of five tissue types: cortical grey matter, subcortical grey matter, white matter, cerebrospinal fluid, and ‘5th type’ (e.g., pathology). The FOD data and the 5TT image were used to generate 120 million streamlines using the anatomically constrained tractography framework (R. E. Smith et al., 2012), using dynamic and the second-order integration over fibre orientation distributions (iFOD2; Tournier et al., 2012) probabilistic fibre-tracking algorithm, using default MRtrix parameters, with the exception of FOD cutoff 0.06, maximum length 250 mm, step size 1 mm, and backtrack specified. This set of streamlines is referred to as the whole-brain-tractogram thereafter.

Specifically, we calculated from a highly curated tractography rendering of the cerebro-cerebellar loop, after thresholding the streamlines to eliminate possible spurious tracts. An average tract obtained from 5 to 10 to 28 subjects, thresholded to represent the group, may lose the finer details of the connectome that are key when using a ∼400 region grey matter parcellation atlas as in this work. On the other hand, the connectome from the union of the tracts, if not thresholded, would inflate this finer connectivity (if thresholded, these connections would be downweighted). The impact of averaging individual subject streamlines on the actual connectome has to be demonstrated in a separate study, as the high variability of the streamlines is likely to correspond to a fairly stable connectome.

The cerebello-thalamo-cortical (CTC) and cortico-ponto-cerebellar (CPC) tracts were extracted from the whole-brain tractogram by using contralateral cerebral and cerebellar cortices, cerebellar peduncles, contralateral red nuclei, and thalami as regions of interest (for more details, see Palesi et al., 2015, 2017). To define the strength of the cerebellar connectivity with each of brain parcel, the log10 of the number of streamlines was used to weight the CTC and CPC tracts (Abos et al., 2019; Palesi et al., 2021). To ensure that the single-subject connectome was representative of the group-level parcellation, we calculated the DICE coefficient between the mean map of both the CTC and CPC tracts (L and R) of a further 27 subjects in MNI space (including only those voxels that were common to at least 70% of subjects, that is, less than the 90,000,000 streamlines used for the individual connectome); the DICE was 0.7, suggesting strong correspondence between our single subject (who preserved the fine-scale nature of the connectome) and the group template.

### Cortico-Cerebellar Activity Flow Mapping

To determine whether cortico-cerebellar interactions could transform cortical or cerebellar task-evoked activity into respective cerebello-cortical task activity, we modified the activity flow mapping procedure (Cole et al., 2016) to incorporate estimates of cortico-cerebellar (CPC) and cerebello-cortical (CTC) structural connectivity. Specifically, for each trial type, block and subject, we calculated:

$${\text{A}}_{CTX}=W_{CBM}^{t}\cdot CPC$$ (4)

$${\text{A}}_{CBM}=W_{CTX}^{t}\cdot CTC$$ (5)

where *W*_*t*_ is the evoked response estimate for every cortical (*W*_*CTX*_) or cerebellar (*W*_*CBM*_) parcel, CPC and CTC are the structural connectivity matrices described above, and A_*CTX*_ and A_*CBM*_ are the predicted activity pattern for each subgroup. For each trial type, block and subject, the predicted cortical and cerebellar activity patterns were then empirically compared to the observed activity patterns using Pearson correlations. A series of *t* tests were used to compare the Pearson’s correlation loadings, with the nonmatching predictions (e.g., using the cortical BOLD for balance trials to predict cerebellar BOLD for calculation trials) used a simple null model that contained all the same spectral features but spatiotemporal sequences that did not match the data. Finally, we created separate null distributions following a random permutation (Nichols & Holmes, 2002) of both CPC and CTC, separately (each with 5,000 iterations).

## ACKNOWLEDGMENTS

Data were provided by the Human Connectome Project, WU-Minn Consortium (PI: David Van Essen and Kamil Ugurbil; 1U54MH091657) funded by the 16 NIH Institutes and Centers that support the NIH Blueprint for Neuroscience Research; and by the McDonnell Center for Systems Neuroscience, Washington University.

## SUPPORTING INFORMATION

Supporting information for this article is available at https://doi.org/10.1162/netn_a_00308.

## AUTHOR CONTRIBUTIONS

Eli J. Müller: Conceptualization; Investigation; Methodology; Writing – review & editing. Fulvia Palesi: Formal analysis; Methodology; Writing – review & editing. Kevin Y. Hou: Formal analysis; Methodology. Joshua Tan: Writing – review & editing. Thomas Close: Writing – review & editing. Claudia A. M. Gandini Wheeler-Kingschott: Investigation; Writing – review & editing. Egidio D’Angelo: Investigation; Writing – review & editing. Fernando Calamante: Formal analysis; Investigation; Methodology; Writing – review & editing. James Shine: Conceptualization; Data curation; Formal analysis; Investigation; Methodology; Project administration; Resources; Software; Supervision; Validation; Visualization; Writing – original draft; Writing – review & editing.

## FUNDING INFORMATION

James Shine, National Health and Medical Research Council (https://dx.doi.org/10.13039/501100000925), Award ID: 1193857.

## TECHNICAL TERMS

Parallel processing: The running of two or more processes in tandem.
Cerebellum: A physically small but neuronally dense structure in the hindbrain important for sensorimotor adaptation and anticipation.
Cerebral cortex: The thin, outer layer of the telencephalon important for deliberate, conscious processing.
Dual task: The performance of two simultaneous tasks, one of which is typically assumed to be easier to automatise than the other.
Low-dimensional: A system whose activity can be expressed through a smaller number of components without a substantial loss in explained variance.
Integration: The formation of a unified or coordinated whole—in the case of brain networks, the presence of relatively diffuse connections across brain regions.
Segregation: The formation of setting something apart from others—in the case of brain networks, the presence of tight-knit subcommunities.
Automaticity: The ability to perform a behaviour without deliberate, focussed attention.
