# Supplementary material for: Parallel processing relies on a distributed, low-dimensional cortico-cerebellar architecture
Source: Netw Neurosci. 2023 Jun 30;7(2):844–63. doi: 10.1162/netn_a_00308 (PMC10312290; doi:10.1162/netn_a_00308)
Supplement: Supplementary file 1 [file netn-7-2-844-s001.pdf]

Supplementary Figures

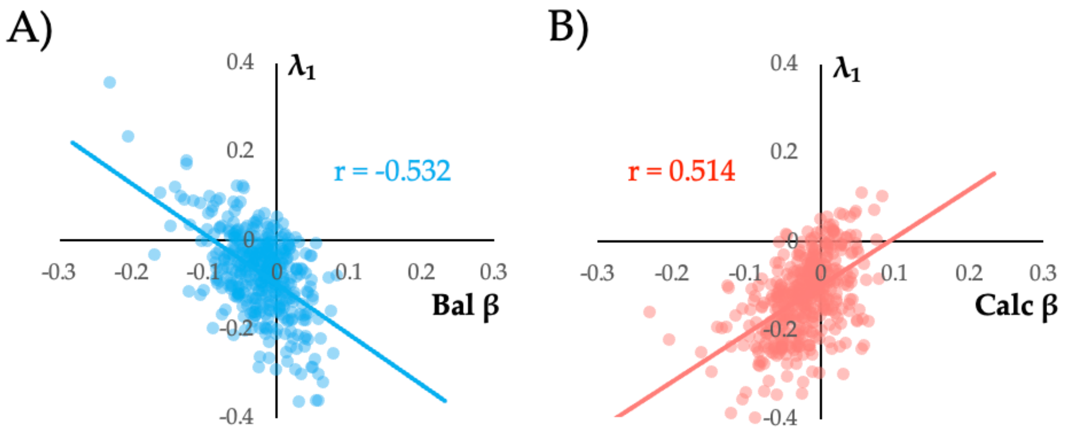

Figure S1 – Correlation between Balance and Calculation maps and leading principal component. A) Loadings of the principal eigenvector (λ_1) in both cerebral cortex (left) and cerebellum (right); F) scatter between mean Balance β map and λ_1 ($r = -0.532$; $p_{SPIN} = 0.002$); G) scatter plot between the mean Calculation β map and λ_1 ($r = 0.514$; $p_{SPIN} < 0.0001$).
